# A nested case-control study on the association of gut virome in early pregnancy and gestational diabetes mellitus

**DOI:** 10.3389/fmicb.2024.1461259

**Published:** 2024-11-15

**Authors:** Xinrui Wu, Xinpeng Liu, Wenbo Xu, Wenhui Chen, Zixin Zhong, Hongzhuan Tan, Tianyu Xiang

**Affiliations:** ^1^School of Medicine, Jishou University, Jishou, China; ^2^Xiangya School of Public Health, Central South University, Changsha, China

**Keywords:** gestational diabetes mellitus, gut virome, nested case-control study, gut microbiome, cross-kingdom correlation, prediction model

## Abstract

**Background:**

Recent studies have increasingly shown the connection between gut microbiome and gestational diabetes mellitus (GDM). However, no studies have explored the relationship between the gut virome and GDM, and the underlying mechanism remains unknown.

**Methods:**

We performed a nested case-control study within a follow-up cohort, enrolling 51 patients with GDM and 51 healthy controls. Shotgun metagenomics sequencing was used to explore gut virome profiles during early pregnancy.

**Results:**

Diversity analysis revealed no difference in the overall gut virome composition between two groups, however, we found greater abundance of *Escherichia phage SH2026Stx1* (*Q* = 0.23), *Enterobacteria phage mEp043 c-1* (*Q* = 0.21), *crAssphage cr50_1* (*Q* = 0.21), *Enterobacteria phage phi80* (*Q* = 0.21), and *Escherichia phage HK106* (*Q* = 0.23) in GDM patients. Cross-kingdom correlation analysis showed the negative correlation between the gut bacterium *Eubacterium eligens* and three bacteriophages (*Escherichia phage SH2026Stx1*, *Enterobacteria phage mEp043 c-1*, and *Escherichia phage HK106*) in GDM group (*r* < 0, *P* < 0.05). Based on gut microbial features and clinical indicators, we constructed a new prediction model using random forest method for GDM with good predictive performance (AUC of 0.893, 95% *CI*: 0.736 ∼ 0.990).

**Conclusion:**

This study is the first to investigate the relationship between the gut virome and GDM as well as the cross-kingdom correlation between gut viruses and bacteria in GDM. Our findings could enhance strategies for preventing and treating GDM from the perspective of gut microbiome, offering valuable insights into its pathogenesis.

## Introduction

Gestational diabetes mellitus (GDM), characterized by glucose intolerance first occurring during pregnancy (Buchanan et al., 2007), is a significant complication affecting 7.0–27.6% of pregnant women worldwide, and its prevalence is increasing (Zhu and Zhang, 2016; Wang et al., 2022). Previous studies found that GDM can lead to adverse pregnancy outcomes, such as obstructed labor, hypertensive disorders of pregnancy, macrosomia and so on (Metzger et al., 2008; Mitanchez, 2010; Billionnet et al., 2017). Furthermore, it significantly raises the risk of cardiovascular diseases, type 2 diabetes (T2D), and mental disorders in both affected pregnant women and their offspring (Dabelea and Pettitt, 2001; Bellamy et al., 2009). Despite extensive researches, the precise pathogenesis of GDM remains poorly understood. Thus, investigating innovative perspectives on the mechanisms is critical for enhancing its prevention and treatment.

The human gut microbiome, comprising bacteria, fungi, viruses, archaea, and protozoa, plays a crucial role in maintaining internal environmental balance and promoting health (Qin et al., 2012). Emerging evidence shows that the human gut microbiome is closely related to GDM. Ma et al. (2020) reported that the gut microbial genera *Tyzzerella 4* and *Eisenbergiella* were positively correlated with fasting blood glucose levels in GDM patients. Furthermore, Mendelian randomization analyses have confirmed the causal relationships involving gut microbiota, their metabolites, and GDM (Wu et al., 2023). Recent studies have also investigated the association between the gut virome and T2D, which shares mechanistic similarities with GDM (Crusell et al., 2018). Rasmussen et al. (2020) found impaired glucose tolerance in mice on a high-fat diet that received fecal virus transplantation. In another study, Fan et al. (2023) assessed the intestinal microorganism profiles of 90 individuals with T2D and 49 healthy controls, revealing significant decreases in *Flavobacterium phage* and *Cellulophaga phaga* in the T2D group. The combined use of gut viral and bacterial markers showed promising diagnostic potential for T2D (Fan et al., 2023).

Viruses, as crucial contributors to intestinal homeostasis, likely play a role in regulating blood glucose metabolism through the gut microbiome (Reyes et al., 2012). Using the mouse model, researchers found that gut virome can activate host immune responses via intestinal mucosa and intestinal immune cells (Gogokhia et al., 2019), which are involved in the pathogenesis of obesity-related insulin resistance and T2D (Esser et al., 2014). Furthermore, phages can also influence the metabolic activity of their hosts through prophage integration and horizontal gene transfer (de Jonge et al., 2022). However, existing studies have some limitations. Firstly, most studies have focused on bacteria, and there has been no research on the relationship between the gut virome and GDM to date. Secondly, animal experiments struggle to replicate the complex environment of the human intestinal tract where diverse microorganisms coexist. Furthermore, most existing findings derive from observational studies, and the temporal relationship between exposure (gut microorganisms) and outcome (GDM) remains unclear for making causal inferences (Smith and Ebrahim, 2002).

In this study, we conducted a prospective nested case-control study to analyze the gut virome of GDM patients in early pregnancy and explore the cross-kingdom correlation between gut viruses and gut bacteria in GDM. Based on the gut microbial features and clinical indices, a new prediction model of early pregnancy GDM was constructed. Our findings could enhance strategies for preventing and treating GDM by considering the microorganisms, and offer novel insights into its pathogenesis.

## Materials and methods

### Study population

Between March 2017 and December 2018, pregnant women from the early pregnancy cohort at the Maternity and Child Health Hospital of Hunan Province were included in our study (no.ChiCTR1900020652). Participants were enrolled during early pregnancy and followed up until 42 days postpartum. Inclusion criteria comprised: (1) singleton pregnancy through natural conception; (2) absence of pre-existing diabetes, hypertension, or thyroid disease prior to pregnancy; (3) no acute infections and no recent antibiotic usage within the preceding 2 weeks. According to the International Association of Diabetes and Pregnancy Study Groups Standard (Metzger et al., 2010), pregnant women were diagnosed with GDM if one or more of the following applied glucose levels were elevated at 24 ∼ 28 weeks in pregnancy (fasting ≥ 5.1 mmol/L, 1 h ≥ 10.0 mmol/L, 2 h ≥ 8.5 mmol/L).

This cohort initially comprised 744 subjects followed from early pregnancy to postpartum, with a dropout rate of 6.0%. After excluding individuals with incomplete clinical data, inadequate stool samples, or unsuccessful metagenomic sequencing, 51 pregnant women diagnosed with GDM were selected for the case group. A control group of 51 pregnant women with normal blood glucose levels was randomly chosen from the original cohort. All participants provided informed written consent. The study was approved by the Maternity and Child Health Hospital of Hunan Province (no. EC201624), and all procedures adhered to applicable guidelines and regulations.

### DNA extraction and metagenomics sequencing

Microbiome DNA was extracted from 180 to 200 mg of feces using the QIAamp Fast DNA Stool Mini Kit (Qiagen, Germany). Extracted DNA were checked by the NanoDrop2000 and Qubit 4.0 for concentration and purity, and stored at −20°C until use. Sequencing libraries were prepared using the NEBNext Ultra™ DNA Library Preparation Kit (Illumina, USA) according to the manufacturer’s instructions. Clustering of index-coded samples was conducted on a cBot Cluster Generation System, followed by shotgun metagenomics sequencing on the Illumina HiSeq platform (paired-end; insert size, 350 bp; read length, 150 bp).

Raw reads were first processed with fastp version 0.23.2, which trimmed reads if adapter-contaminated, N-containing, shorter than 90 bp, or bases with quality low than 15 were more than 50% of reads to get the high quality data (Chen, 2023). Then, reads aligned to the human reference genome (GRCh38/hg38) were removed using Bowtie2 version 2.5.3 to get the clean data (Langmead and Salzberg, 2012). To enhance sequencing depth of virus sequences per sample, virus-related reads in each sample were screened using Kraken2 version 2.1.3 and cross-assembled via MEGAHIT version 1.1.2 with a k-mer ranging from 27 to 127 to construct contigs from all samples (Li et al., 2016; Wood et al., 2019). Contigs longer than 1000 bp were retained then assessed using CheckV version 1.0.3 to test whether their viral gene count was more than the number of microbial genes (Nayfach et al., 2021). Contig coverage, measured in Reads Per Kilobase per Million mapped reads (RPKMs), was calculated using Bowtie2 version 2.5.3 with default parameters. This measurement was normalized based on both contig length and the number of mapped reads in each specimen (Han et al., 2018). The average RPKM for each virus was calculated and normalized across all samples to derive the abundance profile. Additionally, the details on taxonomic identification and abundance profiling of gut bacteria can be found in Supplementary material.

### Statistical analyses

Alpha diversity was measured by Shannon and Simpson indices. Principal coordinates analysis (PCoA), Permutational multivariate analysis of variance (ADONIS), and Analysis of Similarities (ANOSIM) were utilized to cluster and visualize the samples as well as quantify the differences in beta diversity between groups.

Multivariate linear regression analysis (MaAsLin2) was conducted to explore the differential microbial features (gut viruses and bacteria) between groups (Morgan et al., 2012). Taxa with *P* < 0.05 and corrected *P-*value (*Q*-value) < 0.25 were ultimately considered (Glickman et al., 2014). Spearman’s rank correlation assessed correlations between differential gut viruses, clinical indicators, and bacteria, visualized using the corrplot package. To determine the co-occurrence network of bacterial and viral communities in healthy controls and GDM patients, the top 50 most abundant gut viruses and bacteria at the species level were selected, and virus-bacteria linkages were further examined. Spearman correlation analysis was carried out to determine these associations with the *P*-value corrected for multiple testing utilizing the Benjamini-Hochberg FDR method. The cutoffs of the correlation coefficient and the FDR-corrected *P*-value were set at 0.5 and 0.05, respectively. The igraph package was applied to calculate degree distribution, node betweenness, and network natural connectivity.

Two different methods including random forest and logistic regression models were used to predict GDM in early pregnancy, with samples randomly split into training (70%) and validation sets (30%). Random forest model was set as prime method and model performance was evaluated using metrics such as area under curve (AUC), accuracy, recall, precision, F1 score, sensitivity, and specificity and visualized using the ROCR package to generate receiver operating characteristic curves (ROC). These analyses were conducted utilizing R version 4.2.2, with the significance threshold set at *P* < 0.05.

## Results

### Characteristics of the subjects

This study included 51 GDM patients and 51 controls with normal blood glucose levels during pregnancy. Baseline data showed that the average age of GDM patients was 30.58 ± 3.70 years and 30.66 ± 3.83 years for controls, without significant difference. The BMI was significantly higher in the GDM group compared to the control group (*P* = 0.018). Significant differences existed between groups in height, systolic blood pressure (SBP), hemoglobin (HGB), glucose (GLU), triglycerides (TG), and high-density lipoprotein cholesterol (HDL-C) (*P* < 0.05). Furthermore, logistic regression was employed for multivariate analysis to explore determinants related to GDM based on univariate analysis results. Height, HGB, and GLU were associated with GDM occurrence (*P* < 0.05). Additional subject characteristics and results of logistic regression analysis are presented in Table 1 and Supplementary Table 1.

**TABLE 1:** Characteristics of the subjects.

	GDM (*n* = 51)	Controls (*n* = 51)	*P-*value
**Basic characteristics**			
Age (year)	30.58 ± 3.70	30.66 ± 3.83	0.923
Gestational age of fecal sample (weeks)	13.55 ± 0.91	13.71 ± 1.00	0.738
Primipara, *n*(%)	26 (51.0%)	31 (58.8)	0.426
Smoking history, *n*(%)	2 (3.9%)	2 (3.9%)	1.000
Drink history, *n*(%)	3 (5.9%)	3 (5.9%)	1.000
**Anthropometrics factors**			
Waist (cm)	81.02 ± 8.06	79.22 ± 8.09	0.262
Weight (kg)	57.00(8.00)	54.00 (11.00)	0.099
Height (cm)	158.39 ± 3.50	159.97 ± 4.08	0.039*
BMI (kg/cm^2^)	22.60 (3.53)	20.70 (3.21)	0.018*
SBP (mmHg)	119.55 ± 9.42	115.18 ± 10.91	0.033*
DBP (mmHg)	78.00 (11.00)	76.00(9.00)	0.245
**Biochemical parameters**			
HGB (g/L)	128.00 (10.00)	122.00 (12.00)	0.009*
GLU(mmol/L)	4.90 (0.63)	4.60 (0.47)	0.001*
ALB (g/L)	44.95 ± 2.72	44.98 ± 2.72	0.963
TG (mmol/L)	1.62 (0.95)	1.25 (0.62)	0.010 *
TC (mmol/L)	4.57 ± 0.77	4.48 ± 0.67	0.519
HDL-C (mmol/L)	1.74 ± 0.33	1.95 ± 0.34	0.002*
LDL-C (mmol/L)	2.50 (1.10)	2.40 (0.72)	0.244
AST (U/L)	17.20 (10.10)	19.60 (8.20)	0.455
ALT (U/L)	15.80 (18.10)	15.40 (16.30)	0.327

*P < 0.05; GDM, gestational diabetes mellitus; BMI, body mass index; SBP, systolic pressure; DBP, diastolic pressure; HGB, hemoglobin; GLU, glucose; ALB, albumin; TG, triglyceride; TC, total cholesterol; HDL-C, high density lipoprotein cholesterol; LDL-C, low density lipoprotein cholesterol; AST, aspartate amino transferase; ALT, alanine transaminase.

### Metagenomics sequencing analysis

**FIGURE 1:**
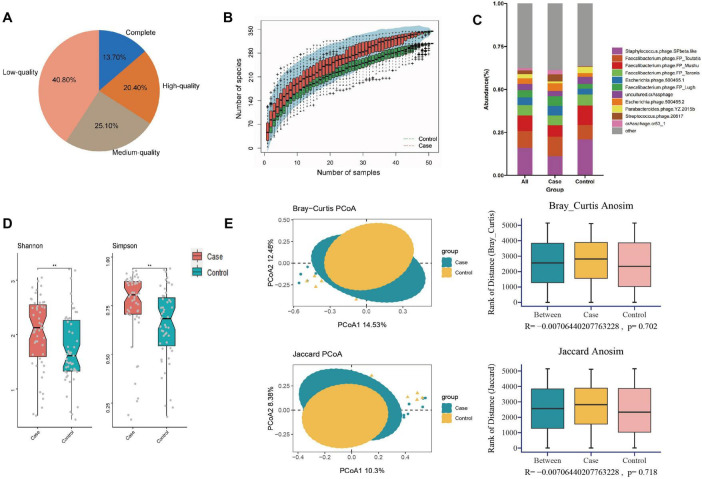
Composition and diversity analysis of gut virome in different groups. **(A)** Pie plot of the quality assessment; **(B)** rarefaction curve; **(C)** composition analysis; **(D)** alpha diversity; **(E)** the PCOA and ANOSIM analysis on beta diversity. **P < 0.01.

In this study, we obtained a total of 778.96 gigabytes of raw data, averaging 82,525 reads per sample after sequencing. The number of reads per sample ranged from 39,822,166 to 137,574,946. Quality control was performed using fastq, resulting in 5,089,345,182 high-quality reads collected for further analysis. After cross-assembling reads from all samples, we obtained a total of 312,465 contigs, averaging 3,063 bp per sample, ranging from 1,000 to 213,528 bp. Based on the quality estimation using the CheckV algorithm (Nayfach et al., 2021), 13.7% of these contigs were evaluated as complete viral genomes, 20.4, 25.1, and 40.8% of them were high-, medium-, and low-quality viruses, respectively (Figure 1A). Rarefaction curves indicated that the sequencing depth adequately reflected the microbial diversity of the samples (Figure 1B).

### Composition and diversity analysis

The contigs of all samples were annotated to viruses at different classification levels (8 orders, 43 families, 176 genera, 328 species). The average number of viruses per sample at different classification levels were 5.22 ± 1.20 orders, 29.67 ± 4.55 families, 98.23 ± 9.25 genera, and 148.34 ± 12.37 species, respectively.

At the species level, the five most abundant taxa included *Staphylococcus phage SPbeta-like* (16.10%), *Faecalibacterium phage FP Toutatis* (9.78%), *Faecalibacterium phage FP Mushu* (8.99%), *Faecalibacterium phage FP Taranis* (6.11%), *Escherichia phage 500465-1* (4.44%), collectively constituting about 45.42% of all gut viruses. Figure 1C demonstrated the top 10 viruses among all participants, including the GDM and control groups. To facilitate subsequent cross-kingdom analysis and prediction model construction, we additionally annotated the gut bacteria of all participants. A total of 1,303 species of bacteria were found at the species level, of which 62 species were common with relative abundance greater than 0.10%, accounting for 61.02% of the total gut bacteria (Wang and Jia, 2016).

Analysis of alpha diversity revealed that women who later developed GDM exhibited significantly higher diversity (higher Shannon, *P* = 0.007; Simpson, *P* = 0.003) compared to controls (Figure 1D and Supplementary Table 2). PCoA plots based on Bray-Curtis and Jaccard distances were constructed to detect the differences in beta diversity, while both ADONIS and ANOSIM analyses indicated no differences in the overall composition of the gut virome between the two groups (Figure 1E and Supplementary Table 3).

### Differential gut microbiome and correlation analysis

**FIGURE 2:**
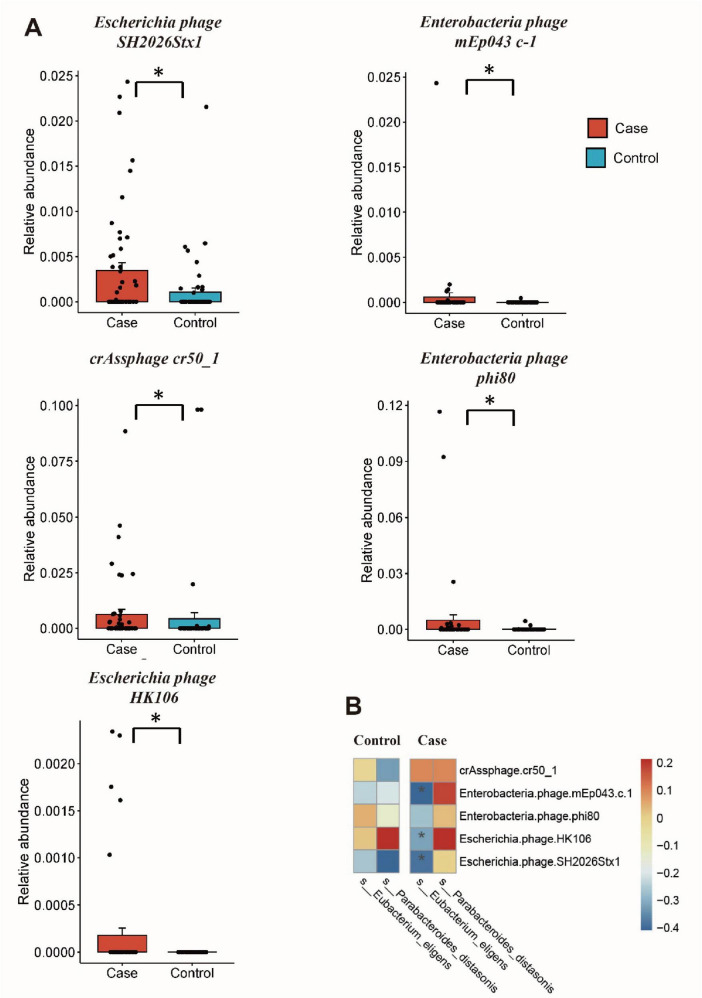
Differential gut microbiome and correlation analysis. **(A)** The relative abundance of differential gut viruses using MaAsLin2 analysis; **(B)** cross-kingdom correlation analysis of the differential viruses and bacteria. *Q < 0.25 and *P* < 0.05 in panel **(A)**, *P* < 0.05 in panel **(B)**.

The MaAsLin2 analysis was conducted to investigate differences in the gut microorganisms between GDM patients and healthy controls. For gut viruses, there were increased abundance of *Escherichia phage SH2026Stx1* (MaAsLin coefficient = 16.72, *Q* = 0.23), *Enterobacteria phage mEp043 c-1* (MaAsLin coefficient = 15.41, *Q* = 0.21), *crAssphage cr50_1* (MaAsLin coefficient = 11.52, *Q* = 0.21), *Enterobacteria phage phi80* (MaAsLin coefficient = 7.07, *Q* = 0.21), and *Escherichia phage HK106* (MaAsLin coefficient = 5.23, *Q* = 0.23) in GDM patients. While, for gut microbiota, there were less *Eubacterium eligens* (MaAsLin coefficient = −3.35, *Q* = 0.04) and *Escherichia phage HK106* (MaAsLin coefficient = −2.75, *Q* = 0.04) in GDM patients. Even after adjusting for potential confounding factors such as age and gestational age, significant differences in these species persisted between the two groups (Figure 2A and Supplementary Table 4).

Spearman’s rank correlation showed that the relative abundance of *Enterobacteria phage phi80* is negatively correlated with GLU (*r* = −0.217, *P* = 0.028), while other differential gut viruses showed no statistically associations with clinical factors (Supplementary Figure 1 and Supplementary Table 5). Subsequently, the correlation between differential viruses and bacteria was analyzed, revealing a negative correlation between *Eubacterium eligens* and three bacteriophages (*Escherichia phage SH2026Stx1*, *Enterobacteria phage mEp043 c-1*, and *Escherichia phage HK106*) in GDM group (*r* < 0, *P* < 0.05) (Figure 2B and Supplementary Table 6). However, no such correlation was observed in the control group.

### Virus-bacteria co-occurrence network analysis

**FIGURE 3:**
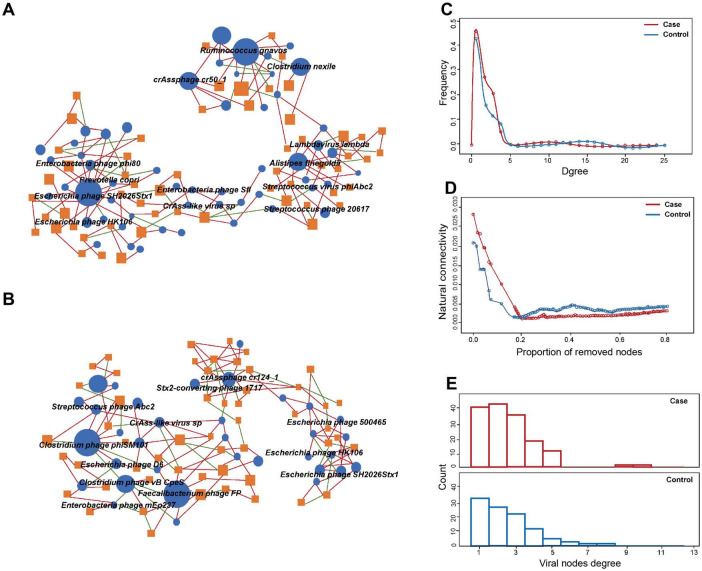
Comparison of virus-bacteria co-occurrence networks between two groups. Virus-bacteria co-occurrence networks for panels **(A)** case and **(B)** control groups. The nodes are viruses and bacterial species. Blue ellipses and orange rectangles represent viruses and bacteria, respectively. Edges represent positive associations (red) and negative associations (green) between virus and bacteria at the species level. **(C)** Degrees of distribution of virus-bacteria co-occurrence networks. **(D)** Alternations of virus-bacteria co-occurrence networks based on the proportion of removed modes. **(E)** Degrees of viral nodes degree in virus-bacteria co-occurrence networks.

Co-occurrence network analysis was used to evaluate the cross-kingdom correlation between gut viruses and bacteria. We observed that *Escherichia phages*, including *Enterobacteria phage phi80*, *Escherichia phage SH2026Stx1*, and *Escherichia phage HK106*, were positively correlated with *Prevotella copri*, which suggested that *Prevotella copri* could infect these species (Figure 3A). We also found that *Clostridium phage phiSM101* and *Clostridium phage vB CpeS* were more abundant in control group, and multiple viruses, including *Escherichia phage D6*, were negatively associated with them (Figure 3B). The results showed the predominance of positive correlations over negative ones in both groups (*P* < 0.05).

Structurally, the network of GDM cases comprised 98 nodes and 163 edges, while that of controls consisted of 77 nodes and 135 edge. The densities of the networks in these two groups were 0.0283 and 0.0237, respectively. Degree distribution of the GDM and healthy control network was different (Figure 3C). Despite the higher number of nodes and edges in the network of GDM cases, the network natural connectivity of which was more fragile than the control group with up to 25% nodes removed (Figure 3D). Specifically, virus-bacteria linkages increased from 33 in control group to 40 in case group, suggesting the linkage is more pervasive in GDM patients than healthy pregnant women (Figure 3E).

### GDM prediction models

We employed both random forest and logistic regression methods to build prediction models for GDM, integrating differential clinical indicators, gut viruses, and gut bacteria. 70% of the subjects were used as the training set, and 30% were used as the validation set for model rehearsal to select the optimal model.

Model 1: We first constructed a predictive model for GDM by using the significantly different clinical indicators. For random forest method, the ROC curve indicated an AUC of 0.742 (95% CI: 0.528 ∼ 0.912). Importance analysis was performed on the model’s included indicators using the Mean Decrease Gini coefficient. The result showed that GLU, HGB, and height were ranked in order of decreasing importance. As for logistic regression model, the ROC curve indicated an AUC of 0.772 (95% CI: 0.663 ∼ 0.891).

Model 2: On the basis of Model 1, two differential gut bacteria in early pregnancy were incorporated into Model 2 as predictive indicators. The ROC curve indicated an AUC of 0.733 (95% CI: 0.515 ∼ 0.902). The ranking of importance was as follows: *Eubacterium eligens*, GLU, *Parabacteroides distasonis*, HGB, and height.

Model 3: Based on Model 1, five differential gut viruses present in early pregnancy were incorporated into Model 3 as predictive indicators. The ROC curve indicated an AUC of 0.769 (95% CI: 0.473 ∼ 0.931). The ranking of importance was as follows: GLU, HGB, height, *crAssphage cr50_1*, *Escherichia phage SH2026Stx1*, *Enterobacteria phage phi80*, *Enterobacteria phage mEp043 c-1*, and *Escherichia phage HK106*.

**FIGURE 4:**
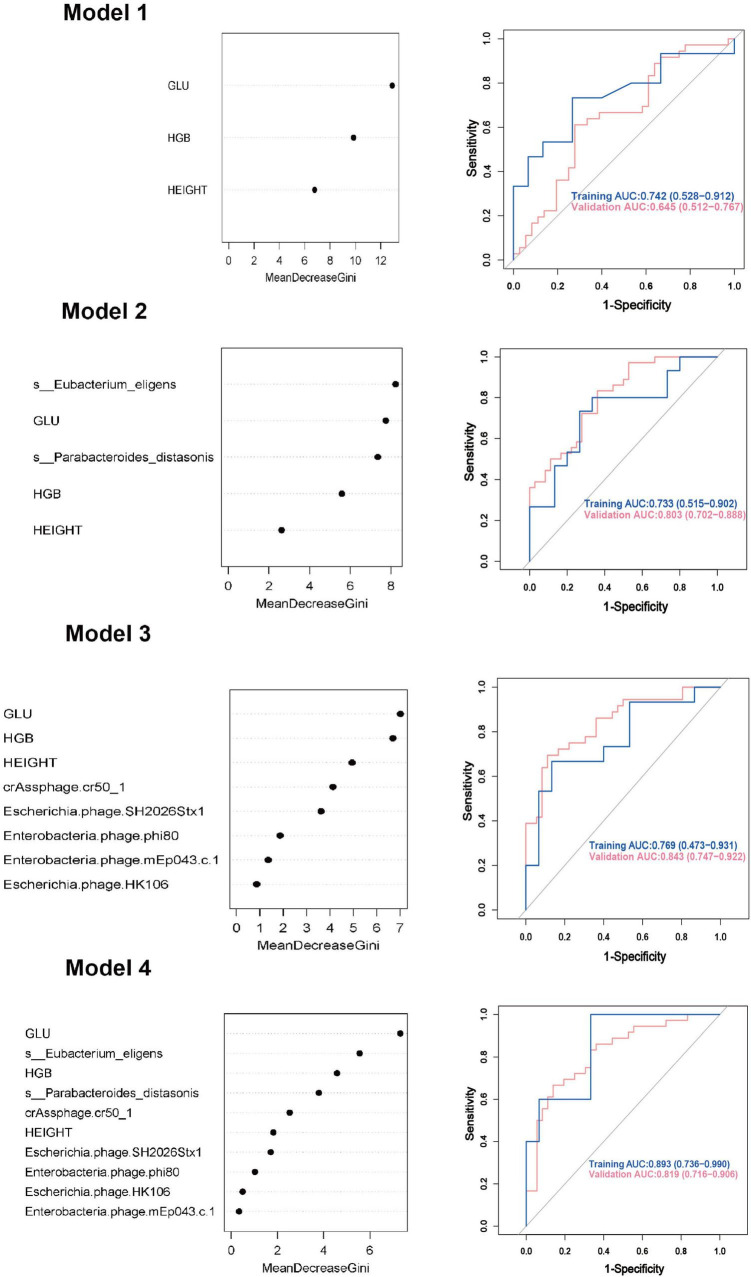
Gestational diabetes mellitus prediction models in early pregnancy using random forest method. GLU, glucose; HGB, hemoglobin; AUC, area under curve; CI, confidence interval.

Model 4: Based on Model 1, seven differential gut microbial species were all added into the Model 4 as predictive indicators. The order of importance ranking was GLU, *Eubacterium eligens*, HGB, *Parabacteroides distasonis*, *crAssphage cr50_1*, height, *Escherichia phage SH2026Stx1*, *Enterobacteria phage phi80*, *Enterobacteria phage*, and *Escherichia phage HK106mEp043 c-1*, indicated an AUC of 0.893 (95% CI: 0.736 ∼ 0.990). The accuracy (0.878), recall (0.941), precision (0.800), and F1 score (0.865) were higher than those of Models 1, 2, and 3. As for logistic regression model, the ROC curve indicated an AUC of 0.930 (95% CI: 0.872 ∼ 0.991). Additionally, the accuracy (0.872), sensitivity (0.861), and specificity (0.891) were higher than those of Models 1, 2, and 3 (Figure 4, Table 2, and Supplementary Figure 2).

**TABLE 2:** Gestational diabetes mellitus prediction models in early pregnancy.

Model	Random forest model					Logistic regression model			
	**AUC (95% CI)**	**Accuracy**	**Recall**	**Precision**	**F1 score**	**AUC (95% CI)**	**Accuracy**	**Sensitivity**	**Specificity**
Model 1	0.742 (0.528, 0.912)	0.718	0.667	0.778	0.718	0.772 (0.663, 0.891)	0.751	0.832	0.672
Model 2	0.733 (0.515, 0.902)	0.763	0.789	0.750	0.769	0.881 (0.801, 0.963)	0.822	0.889	0.743
Model 3	0.769 (0.473, 0.931)	0.774	0.846	0.688	0.759	0.849 (0.772, 0.942)	0.772	0.781	0.769
Model 4	0.893 (0.736, 0.990)	0.878	0.941	0.800	0.865	0.930 (0.872, 0.991)	0.872	0.861	0.891

It shows the results in the training dataset; GDM, gestational diabetes mellitus; AUC, area under curve; CI, confidence interval.

## Discussion

In this nested case–control study, we conducted shotgun metagenomics sequencing of a total of 102 stool samples to characterize and analyze the differences in the composition of gut virome between GDM patients and healthy controls. We investigated the cross-kingdom correlation between gut viruses and bacteria, and developed a novel prediction model for early pregnancy GDM using gut microbial features and clinical indexes, demonstrating robust predictive performance.

In our study, the alpha diversity of gut virome in GMD was higher than that of the control group, consistent with the finding of Ma et al. (2018). However, beta diversity did not show significant differences, suggesting that it may not be the entire composition of the gut virome but rather specific taxa that affect GDM. To explore this possibility, we performed MaAsLin2 analysis and identified five differentially abundant virus taxa in pregnant GDM patients, including four *Enterobacteria phages* and *crAssphage cr50_1*. Our result showed that after adjusting for confounding factors such as age and gestational age, *Enterobacteria phage phi80* was enriched in the GDM patients. A similar difference was observed in patients with colorectal cancer (Zuo et al., 2022). Certain intestinal microbes may increase intestinal permeability and the likelihood of harmful intestinal metabolites entering the bloodstream, thereby affecting systemic inflammation (Abdullah et al., 2021). The inflammatory environment may also promote insulin resistance and β-cell dysfunction, contributing to the onset of GDM (Pinto et al., 2023). Another case-control study identified significant increases in *Enterobacter* and its bacteriophages in patients with T2D, along with elevated levels of serum LPS, IL-6 and TNF-α (Chen et al., 2020). Enrichment of *Enterobacteria phage phi80* might increase circulating LPS levels, thereby inducing systemic subclinical inflammation and affecting insulin sensitivity, potentially contributing to GDM (Mehta et al., 2010).

Additionally, we found significantly elevated levels of *Escherichia phage HK10* and *Enterobacteria phage mEp043 c-1* in the GDM group and those of which were also negatively correlated with the relative abundance of gut bacterium *Eubacterium eligens*. As a short-chain fatty acid (SCFA) butyrate-producing bacteria, *Eubacterium* has been reported to play an important role in maintaining the integrity of intestinal barrier, blood glucose response, and cholesterol homeostasis (Forslund et al., 2015). SCFA can improve insulin sensitivity and satiety by inhibiting HDAC-mediated reprogramming of pancreatic β-cells (Khan and Jena, 2015). This implies that increased levels of *Escherichia phage HK106* and *Enterobacteria phage mEp043 c-1* may reduce the levels of *Eubacterium eligens* (Shuwen and Kefeng, 2022), leading to butyrate depletion in the intestine and initiating a cascade of pro-inflammatory reactions, thus impacting the blood glucose metabolism of pregnant women.

Consistent with our results, Ma et al. (2018) and Chen et al. (2020) reported the increased cross-kingdom correlations between enteric viruses and bacteria in T2D patients. Furthermore, the positive correlations are significantly more than the negative correlations. Yang et al. (2021) found the intensive cross-kingdom correlations in lean controls compared to obese subjects with T2D, which contradicted our findings. The conflicting results are likely due to the limited sample size and sample heterogeneity. The evidence suggests that gut viruses and bacteria are likely to exhibit synergistic or mutually reinforcing relationships, influencing glucose and lipid metabolism. However, additional functional experiments and randomized controlled trials are needed to confirm their association with GDM.

We first developed the model to predict early pregnancy GDM using clinical indicators associated with GDM and achieved an AUC of 0.724, similar to other published GDM prediction models (AUC range: 0.672 ∼ 0.784) (Zheng et al., 2019; Gao et al., 2020; Duo et al., 2023). At present, most of the prediction models for GDM were based on clinical indicators and maternal characteristics. However, recent studies increasingly suggest the potential of gut viral and bacterial markers in diagnosing T2D (Fan et al., 2023; Dash et al., 2023). Adding seven GDM-related gut microbial features (five gut viruses and two bacteria) to the initial model increased the AUC to 0.895 and recall rate to 0.941, respectively. Pinto et al. (2023) established a GDM prediction model incorporating early pregnancy gut microbiome composition, cytokine levels, medical history, and dietary factors, achieving an AUC of 0.830. Ma et al. (2020) and Hu et al. (2021) also constructed the model with AUC of 0.771 and 0.736, respectively. Comparing to the exsiting models only using gut microbiota and other indicators, our study is the first to include gut viruses in the GDM prediction model and demonstrated better prediction performance, thus indicating a potential association between gut viruses and GDM occurrence in early pregnancy. At the same time, our study used two different methods (random forest and logistic regression methods) to construct the model together with training and validation datasets to perform internal validation. The consistent results ensure the stability of our research. Furthermore, all variables included in this model are in the first trimester of pregnancy. Therefore, this model could predict the probability of GDM better in early pregnancy, helping to target high-risk people. Early intervention in these high-risk people may reduce the risk of GDM and have better application value.

Our study has several strengths. Firstly, it is the first to investigate the relationship between the gut virome and GDM, and to analyze interactions between gut viruses and bacteria. Secondly, the nested case-control design ensures that exposure (gut virome) precedes outcome (GDM). Thirdly, our findings were reinforced by a GDM prediction model incorporating early pregnancy gut viruses, gut bacteria, and clinical indicators, which showed good predictive capability.

There are several limitations in our study. Firstly, the cohort only included pregnant women from Hunan Province, limiting the generalizability of our findings to other populations. Secondly, due to an incomplete database and the lack of standardized procedures for handling virus sequences, some metagenomic sequencing data could not be annotated, which affected the robustness and reliability of our findings. Thirdly, while we observed the relationship between the gut virome and GDM, the function and underlying mechanisms remain unclear. Nevertheless, this study is exploratory and aims to guide future research on gut virome functions and gut virus-bacteria interactions in health and disease.

In conclusion, by conducting a nested case-control study, we characterized the gut virome of GDM patients in early pregnancy and explored the correlation between gut viruses and gut bacteria across kingdoms. Using clinical indicators and gut microbial features, we developed a new predictive model for GDM that demonstrated good predictive performance. Our findings have implications for both the prevention and treatment of GDM, offering valuable insights into its pathogenesis. In the future, the gut viruses and bacteria associated with GDM discovered in this study would be validated through function annotation and mouse experiments. Additionally, metabolites derived from gut microbiome would be analyzed to clarify the connections between metagenomics, metabolomics, and GDM, with the goal of thoroughly investigating its pathogenesis and creating early prediction models using a multi-omics approach.

## Data Availability

The datasets presented in this study can be found in online repositories. The names of the repository/repositories and accession number(s) can be found in this article/Supplementary material.
