# Supplementary material for: A nested case-control study on the association of gut virome in early pregnancy and gestational diabetes mellitus
Source: Front Microbiol. 2024 Nov 15;15:1461259. doi: 10.3389/fmicb.2024.1461259 (PMC11604603; doi:10.3389/fmicb.2024.1461259)
Supplement: Supplementary file 2 [file Data_Sheet_1.docx]

**Supplementary Materials**

**A nested case-control study on the association of gut virome in early pregnancy and gestational diabetes mellitus**

Taxonomic Identification and abundance profiling of bacteria

Figure S1. Correlation analysis of the differential gut viruses and clinical indicators.

Figure S2. GDM prediction models in early pregnancy using logistic regression method.

**Taxonomic identification and abundance profiling of bacteria**

To obtain the bacterial composition of fecal samples, firstly, we removed the reads that mapped to viral sequences with Bowtie2 from each sample’s clean data to construct the dataset of these reads (Langmead and Salzberg, 2012). Secondly, these datasets were used to profile the community structure of samples and bacteria information with MetaPhlAn4 using the option “–ignore_viruses,” “–ignore_eukaryotes,” and “–ignore_archaea” (Blanco-Míguez et al., 2023). Finally, the bacterial composition of samples was merged with the “merge_metaphlan_tables.py” command.

**References**

Blanco-Míguez, A.,Beghini, F.,Cumbo, F.,McIver, L. J.,Thompson, K. N.,Zolfo, M., et al. (2023). Extending and improving metagenomic taxonomic profiling with uncharacterized species using MetaPhlAn 4. Nat Biotechnol. 41: 1633-1644. doi: 10.1038/s41587-023-01688-w.

Langmead, B. and Salzberg, S. L. (2012). Fast gapped-read alignment with Bowtie 2. Nat Methods. 9: 357-9. doi: 10.1038/nmeth.1923.


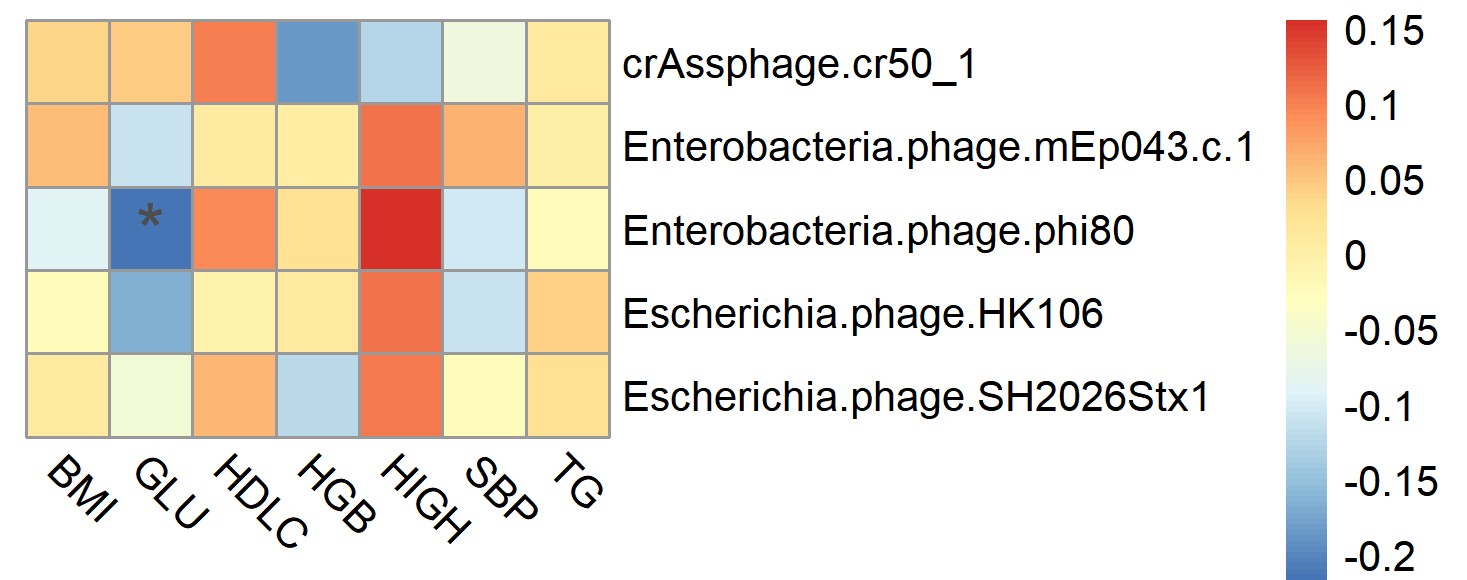


**Figure S1.** Correlation analysis of the differential gut viruses and clinical indicators. *: *P* < 0.05; GLU, glucose; SBP, systolic pressure; HGB, hemoglobin; TG, triglyceride; HDL-C, high density lipoprotein cholesterol.


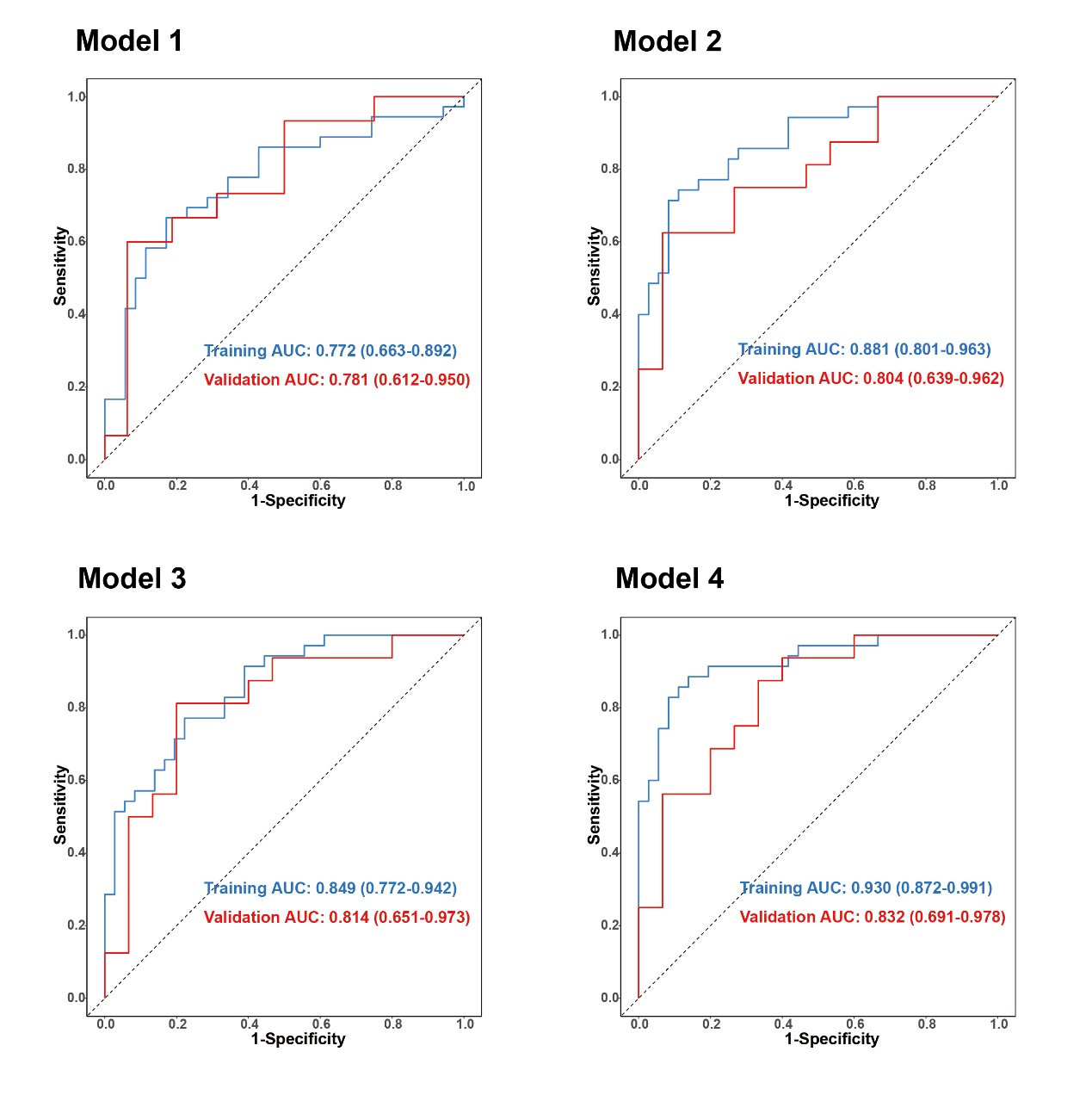


**Figure S2.** GDM prediction models in early pregnancy using logistic regression method. GLU, glucose; HGB, hemoglobin; AUC, area under curve; CI, confidence interval..
